# The Risk Analyses of Lymph Node Metastasis and Recurrence for Submucosal Invasive Colorectal Cancer: Novel Criteria to Skip Completion Surgery

**DOI:** 10.3390/cancers14030822

**Published:** 2022-02-06

**Authors:** Takanori Ozeki, Takaya Shimura, Tomonori Ozeki, Masahide Ebi, Hiroyasu Iwasaki, Hiroyuki Kato, Shingo Inaguma, Yusuke Okuda, Takahito Katano, Hirotada Nishie, Satoru Takahashi, Hiromi Kataoka

**Affiliations:** 1 Department of Gastroenterology and Metabolism, Nagoya City University Graduate School of Medical Sciences, Nagoya 467-8601, Japan; takacpv@yahoo.co.jp (T.O.); hiwasaki@med.nagoya-cu.ac.jp (H.I.); okuda10@med.nagoya-cu.ac.jp (Y.O.); takatano@med.nagoya-cu.ac.jp (T.K.); nishix589998@gmail.com (H.N.); hkataoka@med.nagoya-cu.ac.jp (H.K.); 2 Department of Gastroenterology, Aichi Medical University, Nagakute 480-1195, Japan; tomonoriozeki@gmail.com (T.O.); mebi@aichi-med-u.ac.jp (M.E.); 3 Department of Experimental Pathology and Tumor Biology, Nagoya City University Graduate School of Medical Sciences, Nagoya 467-8601, Japan; h.kato@med.nagoya-cu.ac.jp (H.K.); inaguma@aichi-med-u.ac.jp (S.I.); sattak@med.nagoya-cu.ac.jp (S.T.)

**Keywords:** colorectal cancer, endoscopic submucosal dissection, neoplasms metastasis, recurrence, surgery

## Abstract

**Simple Summary:**

Completion surgery is recommended for patients with submucosal invasive colorectal cancer (pT1 CRC) with known risk factors for lymph node metastasis (LNM). However, completion surgery would be able to be skipped for more of the population with very low risk for LNM and recurrence. The present study thus analyzed both short- and long-term outcomes for high-risk pT1 CRC patients who underwent surgery, showing that lymphovascular invasion was a potential independent risk factor for LNM, and rectal cancer and undifferentiated histology were independent risk factors for poor relapse-free survival in patients with high-risk pT1 CRC. No LNMs were observed in pT1 CRCs with an SM invasion depth ≤2000 µm that had no other risk factors except for budding. Based on the results, novel criteria to skip completion surgery for high-risk pT1 CRC have been established in the current study, which were also validated in an independent validation cohort.

**Abstract:**

(1) Background: Additional surgical resection after endoscopic resection (ER) is recommended for patients with submucosal invasive colorectal cancer (pT1 CRC) who have risk factors for lymph node metastasis (LNM) (high-risk pT1 CRC). This study aimed to identify risk factors for LNM and metastatic recurrence and to determine the low-risk population for whom additional surgery can be omitted among high-risk pT1 CRCs. (2) Methods: We retrospectively identified 404 patients with pT1 CRC who underwent ER or surgery, and patients were divided into three groups: low-risk (*n* = 79); high-risk pT1 with ER (*n* = 40); and high-risk with surgery (*n* = 285). We also enrolled another 64 patients with high-risk pT1 CRC in an independent validation cohort. (3) Results: In the high-risk with surgery group, LNM was seen in 11.2%, and vascular and lymphatic invasions were significantly independent risk factors for LNM on multivariate analysis. No LNMs were observed in pT1 CRCs with a negative vertical margin and SM invasion depth ≤2000 µm that had no other risk factors except for budding. Five patients developed metastatic recurrence in the high-risk with surgery group, and rectal cancer and undifferentiated histology were significantly independent risk factors for poor relapse-free survival. No LNM or recurrent cases were seen in high-risk pT1 CRCs that met these criteria: differentiated adenocarcinoma, no lymphovascular invasion, colon cancer, SM invasion depth ≤2000 μm, and a negative vertical margin, which were validated in an independent validation cohort. (4) Conclusions: Completion surgery may be skipped for high-risk pT1 CRCs that meet our proposed criteria.

## 1. Introduction

Colorectal cancer (CRC) is the third most common malignancy and the second leading cause of cancer deaths worldwide [1]. Recent developments in endoscopic imaging and diagnostic techniques have contributed to an increased discovery of early-stage CRC, including intramucosal (pTis) and submucosal (pT1) CRCs. pTis CRC is a definite indication for endoscopic resection (ER) due to having almost no risk of lymph node metastasis (LNM) when en bloc resection is possible [2,3]. In patients with pT1 CRC, CRC with any of the following findings is considered a surgical indication because of the high risk for LNM (high-risk pT1 CRC) [2]: positive vertical margin after ER; submucosal (SM) invasion depth ≥1000 μm; lymphovascular invasion; undifferentiated histology including poorly differentiated adenocarcinoma, signet-ring cell carcinoma, and mucinous carcinoma; and budding (BD) ≥grade 2 at the site of deepest invasion [4]. Conversely, pT1 CRC without any above-mentioned risk factors is an indication for ER [2,5,6].

However, recent studies have reported low-LNM-risk populations in pT1 CRC with a surgical indication according to the Japanese Society for Cancer of the Colon and Rectum (JSCCR) guideline [2]. Incidence of LNM was very low (1.2%) when patients with pT1 CRC had only deep SM invasion as a risk factor and did not have lymphovascular invasion, undifferentiated histology, or BD as risk factors [7]. Similarly, the incidence of LNM was much lower in patients with pT1 CRC without risk factors except deep SM invasion (1.9%) than in patients with pT1 CRC with risk factors except deep SM invasion (15.8%) [8]. Moreover, additional surgical treatment after ER did not confer a significant benefit in patients with pT1 CRC with a risk factor of only SM invasion (recurrence rate: 2.3% in ER followed by surgery and 3.4% in ER alone), whereas additional surgery significantly decreased the risk of recurrence in patients with pT1 CRC with any other risk factors except SM invasion (recurrence rate: 5.8% in the ER followed by surgery and 58.0% in ER alone) [8]. These results suggest that there are some low-risk populations in the currently defined high-risk pT1 CRCs. It is thus important to identify the population with a very low incidence of LNM and recurrence in patients with high-risk pT1 CRC, which can help avoid unnecessary surgery through endoscopic follow-up instead of completion surgery for that low-risk population. Therefore, we need more data related to the risk of LNM and metastatic recurrence for patients with high-risk pT1 CRC.

Based on this background, this study analyzed both short- and long-term outcomes for patients with high-risk pT1 CRC and proposed new criteria to avoid completion surgery for high-risk pT1 CRCs.

## 2. Materials and Methods

### 2.1. Patients

The data for patients who underwent surgery or ER for CRC from 2003 to 2019 were retrospectively reviewed using the computerized database at Nagoya City University Hospital and Aichi Medical University. Consecutive patients with pT1 CRC who were treated with ER, ER followed by additional surgery, or primary surgery were enrolled in this study. Exclusion criteria were patients with metachronous cancer or previous cancer history, patients with preoperative chemotherapy or radiotherapy, and patients who had no tissue samples. In addition, we also retrospectively enrolled consecutive high-risk pT1 CRC patients with the same criteria in the validation cohort, who were treated from 2019 to 2020 at Nagoya City University and Aichi Medical University. The study protocol conformed to the ethical guidelines of the 1975 Declaration of Helsinki (6th revision, 2008) and was approved by the Institutional Review Board. Written general consent that included the research use of clinical data was obtained from all participants.

### 2.2. Treatment

ER, including endoscopic mucosal resection (EMR) and endoscopic submucosal dissection (ESD), was performed for the lesions when the endoscopists determined them to be endoscopically resectable tumors. EMR was performed using intramucosal saline injection followed by snaring. The VIO 300D (ERBE Elektromedizin GmbH, Tubingen, Germany) was used as the electrosurgical system, and the FlushKnife BT-S (DK2620J; Fujifilm Medical Co., Ltd., Tokyo, Japan); DualKnifeJ (KD-655Q; Olympus Medical Systems Corp., Tokyo, Japan); and SB Knife Jr. (MD-47703W; Sumitomo Bakelite Co., Ltd., Tokyo, Japan) were used for ESD. Surgery was performed by laparotomy or laparoscopic surgery with D2 dissection. Follow-up after resection was performed at least once yearly by blood test or enhanced computed tomography and endoscopy, as appropriate.

### 2.3. Definition

Tumor morphology comprised the Paris endoscopic classification [9], and the morphology was divided into three groups in this study: protruded type including 0-Is, 0-Ip, and Is + IIa; flat/flat elevated type including 0-IIa and 0-IIb; depressed type including 0-IIc, 0-IIa + IIc, 0-IIc + IIa, 0-Is + IIc, and 0-IIc + Is. Each specimen was fixed in formalin, sectioned into 2 mm slices, and embedded into paraffin blocks [10]. Each block was thinly sectioned, stained with hematoxylin and eosin, and reviewed for histology, horizontal and vertical margins, SM invasion depth, vascular invasion, lymphatic invasion, budding, and LNM according to the Japanese Classification of Colorectal, Appendiceal, and Anal Carcinoma [11]. No tumors identified at horizontal and vertical margins were defined as HM0 and VM0, respectively; tumors identified at horizontal and vertical margins were defined as HM1 and VM1, respectively; and cases not assessable for tumor involvement at margins were defined as HMX and VMX, respectively. As for measurement of SM invasion depth, when it was possible to identify or estimate the muscularis mucosa, the depth of SM invasion was measured from the lower border of the muscularis mucosa; when this was not possible, the depth of invasion was measured from the surface of the lesion. In pedunculated lesions with a tangled muscularis mucosa, SM invasion depth was measured as the distance between the point of the deepest invasion and the reference line, which is defined as the neck between the tumor head and the stalk; the depth of limited invasion within the head was defined as head invasion, corresponding to SM invasion depth <1000 µm. Lymphatic and vascular invasions were assessed using HE staining, and immunohistochemistry studies were also used to assess lymphatic invasion with anti-D2-40 antibody (DAKO, Santa Clara, CA) and vascular invasion with anti-CD31 antibody (DAKO) as needed, according to our previous description [12]. Positive D2-40 was defined as lymphatic permeation, and positive CD31 without D2-40 was defined as venous permeation.

BD was defined as a cancer cell nest consisting of one or fewer than five cells infiltrating at the invasive margin of the cancer, and it was categorized into grade 1 (0–4 buds), grade 2 (5–9 buds), and grade 3 (10 or more buds). Based on dominant histology, well- and moderately differentiated adenocarcinoma and papillary carcinomas were defined as differentiated histology, and poorly differentiated mucinous adenocarcinoma and signet-ring cell carcinoma were defined as undifferentiated histology.

The pathological stage was determined on the basis of pathological findings after surgery according to the seventh edition of the Union for International Cancer Control tumor–node–metastasis classification [13]. Pathological analyses were performed by independent pathologists who were blinded to clinical outcomes. The high-risk pT1 group was defined as patients who had lesions with any of the following pathological risk factors for LNM based on the 2019 JSCCR Guidelines for the Treatment of Colorectal Cancer: (1) positive vertical margin; (2) SM invasion depth ≥1000 µm; (3) vascular invasion; (4) lymphatic invasion; (5) undifferentiated histology; and (6) BD ≥grade 2 [2]. The low-risk pT1 group was defined as patients with pT1 CRC without any pathological risk factors. When patients had any high-risk pathological factors after ER, additional surgery was generally recommended. However, the final treatment was determined by patients’ or physicians’ choice based on the degree of LNM risk, physical activity, co-morbidity, and age.

Overall survival was defined as the time from the first day of treatment until death or to the last day of follow up. Relapse-free survival (RFS) was defined as the time from the first day of treatment to recurrence, death, or the last day of follow up (depending on which event occurred first).

### 2.4. Statistical Analysis

Data were analyzed using the Mann–Whitney *U* test, chi-squared test, and Fisher’s exact probability test, as appropriate. Kaplan–Meier curves were constructed to analyze OS and RFS, and differences between the two groups were compared with the log-rank test. Multivariate analysis of LNM was performed by logistic regression model using a forward selection method with likelihood ratio to estimate the odds ratio (OR) with 95% confidence interval (CI). The multivariate analyses of OS and RFS were performed with the Cox proportional hazard regression model to estimate the hazard ratio (HR) with 95% CI. All statistical tests were two-sided, and *p* values of <0.05 were considered statistically significant. All statistical analyses were conducted using IBM SPSS Statistics, version 25 (IBM Corp., Tokyo, Japan).

## 3. Results

### 3.1. Patients

**Figure 1:**
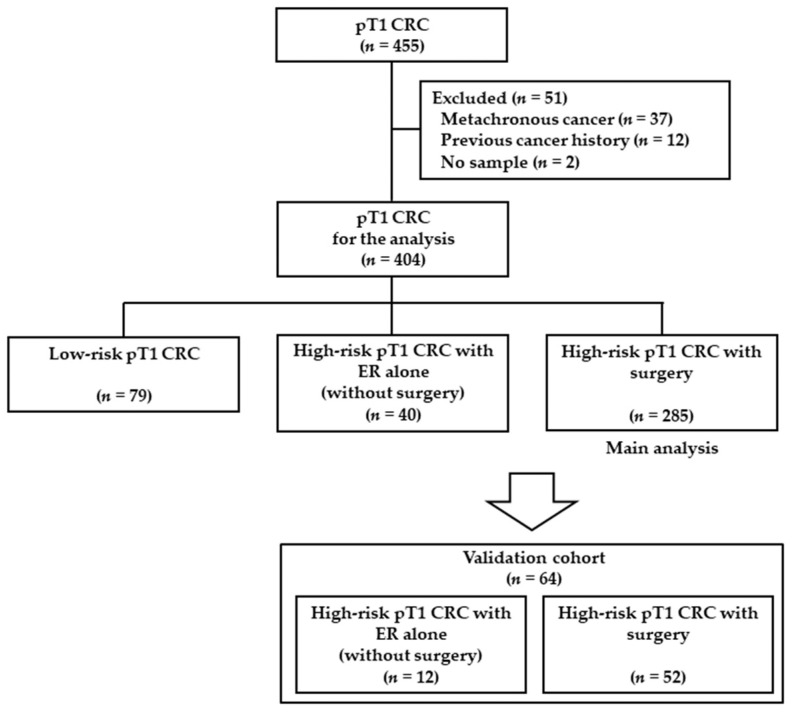
Study flowchart. pT1, pathology stage T1; CRC, colorectal cancer; ER, endoscopic resection.

Among 455 patients with pT1, 51 patients were excluded according to exclusion criteria, and 404 patients were enrolled in the study. The 404 patients with pT1 CRC were divided into three groups based on treatment and pathological risk factors as follows: low-risk pT1 CRC group (*n* = 79); high-risk pT1 CRC with ER alone (without additional surgery) group (*n* = 40); and high-risk pT1 CRC with surgery group (*n* = 285) (Figure 1).

The high-risk pT1 CRC with surgery group included patients who underwent initial surgical resection and additional surgical resection after ER. Patient characteristics are summarized in Table 1. In the low-risk group of patients with pT1 CRC without any risk factors, only 1 patient (1.3%) showed LNM among 24 patients with surgical resection. The lesion with LNM was 30 mm IIa + IIc colon cancer that was initially resected with ESD, and the pathological diagnosis revealed a submucosal invasion depth of 460 μm without any other risk factors. No recurrence occurred in the low-risk group (0/79, 0%). 

As for the high-risk pT1 CRC group, the higher-risk lesions with large size, depressed type, lymphovascular invasion, budding, and a positive vertical margin were significantly more common in the surgery group than in the ER alone group. In the high-risk pT1 CRC with surgery group, 32 patients (11.2%) showed LNM and 5 patients (1.8%) developed recurrence among 285 patients with surgical resection. All patients with recurrence showed metastatic recurrence and did not reveal intraluminal local recurrence. In the high-risk pT1 CRC with ER alone group, there were no patients with metastatic recurrence, but one patient who had a lesion with HMX as the post-ESD histological diagnosis developed an intraluminal residual tumor; the patient underwent curative surgical resection and had revealed no further recurrence thereafter.

In a comparison of OS in patients with ER alone and patients with surgery, no significant difference was found in the low-risk pT1 CRC group, whereas the ER alone showed significantly shorter survival than with surgery in the high-risk pT1 CRC group (Supplementary Figure S1). There were no cancer-related deaths in the high-risk pT1 CRC with ER alone group. Patients with high-risk pT1 CRC with poor physical condition may choose ER alone without additional surgery, but it is not valid because the median follow-up time of the ER alone group (19.1 months) was much shorter than that of the surgery group (46.0 months). Because we cannot assess exact disease stages without pathological assessment using surgically resected tissues in the ER alone group, we decided to mainly analyze the high-risk pT1 CRC with surgery group to identify any risk factors for LNM and recurrence.

### 3.2. LNM

Univariate analysis in patients in the high-risk pT1 CRC with surgery group showed that undifferentiated histology, vascular invasion, or lymphatic invasion are significantly higher in frequency in cases of LNM compared with the absences of these factors. Multivariate analysis identified that vascular and lymphatic invasions were significantly independent risk factors for LNM (vascular invasion, OR: 2.42 (95% CI, 1.06–5.56), *p* = 0.037; lymphatic invasion, OR: 2.64 (95% CI, 1.14–6.09), *p* = 0.024) (Table 2).

According to pathological risk factors based on the JSCCR guidelines, additional analysis was conducted to identify the risk of LNM (Table 3). Interestingly, even if the only risk factor is SM invasion ≥1000 µm with a negative vertical margin, LNM was seen in 4.9% (5/102 patients). Vascular or lymphatic invasion raised the risk of LNM to 14.6% in pT1 CRC with invasion depth ≥1000 µm. BD did not add the risk of LNM (4.2%) in pT1 CRC with invasion depth ≥1000 µm but raised the risk of LNM (21.4%) in pT1 CRC with both risk factors of SM invasion depth and lymphovascular invasion. In addition, both lesions with all pathological risk factors (SM invasion depth, lymphovascular invasion, BD, undifferentiated histology) had LNM. The incidence of LNM stratified according to SM invasion depth was analyzed in the high-risk pT1 CRC with surgery group (Supplementary Table S1). No LNMs were observed in pT1 CRCs with a negative vertical margin and SM invasion depth ≤2000 µm that had no other risk factors except for BD. On the other hand, pT1 CRCs with a positive vertical margin or lymphovascular invasion had a certain risk of LNM even if the pT1 CRCs fulfilled SM invasion depth ≤2000 µm, differentiated histology, and negative BD.

### 3.3. Recurrence

Next, we conducted additional analyses to identify the risk factors of metastatic recurrence in the high-risk group with surgical resection. Univariate analysis showed that tumor location in the rectum, depressed-type morphology, and undifferentiated histology indicated significantly poor RFS. No significant differences were found for the number of examined lymph nodes between colon cancer and rectal cancer (median number (range), colon cancer 9 (0–49) vs. rectal cancer 9 (0–25); *p* = 0.253). Multivariate analysis showed that rectal cancer and undifferentiated histology were significantly independent risk factors for predicting poor RFS (rectal cancer, HR: 2.91 (95% CI, 1.01–8.40), *p* = 0.049; undifferentiated histology, HR: 9.43 (95% CI, 1.17–7.69), *p* = 0.035) (Table 4). As shown in Supplementary Figure S2, 5-year RFS for rectal cancer (85.5%) was significantly shorter than that for colon cancer (95.1%) (*p* = 0.032). In addition, 5-year RFS for undifferentiated histology (50.0%) was also significantly lower than that for the differentiated histology (93.6%) (*p* = 0.005). Conversely, no risk factors were found for OS, including tumor location and morphology (Supplementary Table S2 and Figure S3).

Table 5 shows the breakdown of 5 patients with metastatic recurrence from a total of 404 patients. All 5 patients belong to the high-risk pT1 with surgery group. Median time to recurrence was 30 months (range, 18–56 months). Of these five patients with recurrence, four had rectal cancers, and all five had depressed-type lesions. Interestingly, the only risk factor for one patient was SM invasion depth (2600 µm), although the patient nevertheless developed lung metastasis. Notably, LNM was seen in two patients, but the other three patients had no LNM on pathological assessment of surgically resected specimens. The numbers of examined lymph nodes in the three patients without LNM were 1, 10 and 10 lymph nodes, respectively. As for recurrent findings, one patient developed local LNM and underwent subsequent surgery but developed distant metastasis. The other four patients developed distant metastasis as the initial recurrence, and three patients underwent surgical resection, but one patient experienced recurrence again. Finally, two patients (40%) experienced no recurrence after subsequent surgical resection for recurrent metastasis.

### 3.4. Novel Criteria to Skip Completion Surgery

Based on these results, we created a decision tree to predict the presence of LNM and future recurrence in Supplementary Figure S4. When patients with high-risk pT1 CRC met all of the criteria of differentiated adenocarcinoma, no lymphovascular invasion, colon cancer, SM invasion depth ≤2000 μm, and a negative vertical margin, LNM was 0% (0/29) in patients with high-risk CRC with surgery, and recurrence was 0% (0/39) in patients with high-risk CRC with or without surgery (Table 6).

Moreover, our novel criteria were also validated in an independent validation cohort comprising 64 high-risk pT1 CRC patients (12 patients with ER alone and 52 patients with surgery). Patients’ characteristics in the validation cohort were shown in Supplementary Table S3. Of 52 patients in the high-risk pT1 CRC with surgery group, 6 patients (11.5%) had LNM in the resected specimens, and 2 patients (3.8%) developed metastatic recurrence after surgery.

As also shown in the decision tree of Supplementary Figure S5, the high-risk pT1 CRC patients that meet our criteria did not reveal LNM or recurrence in the validation cohort. When combining both cohorts, our criteria could distinguish 37 patients (11.0%) without LNM from 337 patients with high-risk pT1 CRC who underwent surgery (Table 6).

## 4. Discussion

This study provides several interesting and novel findings. Consequently, we establish new criteria to omit additional surgery in patients with high-risk pT1 CRC.

Among risk factors in the JSCCR guidelines, undifferentiated histology and lymphovascular invasion were potential independent risk factors for LNM in high-risk pT1 CRC. In terms of SM invasion depth as a risk factor, of 102 pT1 CRC patients with a risk factor of only deep SM, 5 patients still had LNM (4.9%), which was higher than the previously reported incidence of approximately 1–2% [7,8,14]. In the previous study, additional surgical treatment after ER did not reduce recurrent events in patients with pT1 CRC with a risk factor of only SM invasion [8]. However, the significance of additional surgery may be estimated as low due to selection bias through the retrospective design of the study. In a recent population-based cohort study comprising 5170 pT1 CRCs, the rate of postoperative severe complication with reintervention was 8.3%, and postoperative mortality was 1.7%; male gender, ASA grade III-IV, cardiac co-morbidity, previous abdominal surgery, open surgery, and subtotal colectomy were the risk factors for postoperative severe complication [15]. At the least, the 4.9% LNM in this study is not a negligible risk for the general population with curable early-stage cancer. On the other hand, when the high-risk pT1 CRC did not have other risk factors except for SM invasion depth, no pT1 CRCs with SM invasion depth ≤2000 µm had LNMs.

As also shown in the JSCCR guideline [2], BD is one of the well-known risk factors for LNM in pT1 CRCs [4,14]. The latest and largest study established a novel predictive system for LNM of pT1 CRC using an artificial intelligence system, which was superior to the currently used guidelines, but BD was not included in these risk factors [16]. These previous studies analyzed all pT1 CRCs, including both low- and high-risk, whereas our study analyzed LNM for only high-risk pT1 CRCs. As a result, BD was not a significant risk factor for LNM in our study despite borderline significance. The discrepancy related to BD may be due to the difference in study population between the previous studies and our study. Interestingly, BD did not create a synergistical risk of LNM for high-risk pT1 CRCs with only SM invasion depth, whereas it synergistically raised the risk of LNM for high-risk pT1 CRCs with both SM invasion depth and lymphovascular invasion. Hence, the influence on LNM by BD may be weaker than that by other risk factors in high-risk pT1 CRC. Taken together, pT1 CRC with SM invasion depth ≤2000 µm may be a good candidate that can omit additional surgery when the pT1 CRC does not have lymphovascular invasion, an undifferentiated histology, and a positive vertical margin after ER. 

The previous Japanese studies with long-term outcomes have reported LNM in 10.8–12.4%, a 2.6–3.7% recurrence rate, and 5-year RFS/DFS in 95–97% of high-risk pT1 CRCs with surgery, but some studies counted both metastatic and local recurrences [5,8,17,18]. In a recent large-scale European retrospective study, metastatic recurrence excluding local intraluminal recurrence occurred in 3.4% (57/1656) of pT1 CRCs, but this study included both low- and high-risk pT1 CRCs, and some patients did not undergo surgical treatment [19]. Metastatic recurrence is an oncological issue, but local intraluminal recurrence is a technical issue caused by a positive resection margin. In the current study, most high-risk patients with a positive or unclear resection margin underwent additional surgery. As a result, only one patient in the high-risk pT1 CRC with ER alone group developed intraluminal local recurrence. Median time to recurrence (30 months) was slightly longer than the approximate 1–2 years reported previously [8,20]. Presumably, the reason would be that we observed only metastatic recurrence and not intraluminal local recurrence in this study. Hence, the present study focused on metastatic recurrence to assess oncological outcomes. Although the metastatic recurrence rate (1.8%) of our study was slightly lower than that of the previous studies, it would be due to the differences in study population and the definition of recurrence, as mentioned above. Since the rate of LNM and 5-year RFS in our study were within the ranges of these previous studies, our study population might represent the general population [5,8,17,18,19].

Rectal cancer was a potential independent factor for metastatic recurrence, which was also consistent with a previous large-scale study [18]. The previous study reported that the risk of recurrence for pT1 CRC after surgical resection has been related to the number of retrieved lymph nodes [21]. Although we feared that the number of removed lymph nodes may be smaller in rectal cancer than in colon cancer, no significant difference was noted between rectal and colon cancers in our study. These results suggest that the high risk of recurrence in rectal cancer may be explained by specific biology [22,23].

In addition, undifferentiated histology that is composed of poorly differentiated mucinous adenocarcinoma and signet-ring cell carcinoma was also an independent factor for poor RFS. This feature is worthy of note because few previous studies related to long-term recurrence have analyzed the metastatic recurrence of undifferentiated histology [5,8,18,20]. A previous study analyzed the risk factors of recurrence for pT1 CRC patients who underwent ER followed by surgery, in which no significant factors were identified, but undifferentiated histology revealed the highest HR of 5.3 [95% CI, 0.9–31.5] among all risk factors [8]. Since low-risk pT1 CRCs were included in this analytical cohort and pT1 CRC patients with primary surgery were excluded from this study [8], the obtained results might be slightly different from our study. Undifferentiated histology is a well-established risk factor for recurrence and poor prognosis in stage II CRC [24,25]. Although there is a lack of definite data for pT1 CRC due to low frequency, undifferentiated histology may also be a risk factor for metastatic recurrence.

The depressed type was a risk factor on univariate analysis but was not significant on multivariate analysis. Indeed, all five lesions with recurrence exhibited depressed findings of the tumors in our study. It has been reported that depression was the most significant finding for predicting pathological high-risk factors of the JSCCR guideline [14]. Although the presence of depression may represent malignant features, it is inconclusive for recurrence in the current study.

Interestingly, the risk factors were different between the incidence of LNM and recurrence. Moreover, of five patients with recurrence, only two showed LNM, but the other three patients showed no LNM in surgically resected specimens. These results suggest that LNM does not reflect future recurrence when lymph node dissection is properly conducted. Potentially, LNM could be arrested by surgical resection in patients with pT1 CRC. Conversely, rectal cancer and undifferentiated carcinoma may possess innate malignant potential.

Based on findings in this study, we propose novel criteria to select a relatively low-risk population among patients with currently defined high-risk pT1 CRC. Patients with high-risk pT1 CRC who met all five criteria, (1) differentiated adenocarcinoma; (2) no lymphovascular invasion; (3) colon cancer; (4) SM invasion depth ≤2000 μm; and (5) negative vertical margin, did not show LNM or experience recurrence in this study. Importantly, en bloc resection is necessary for precise pathological assessment. These novel criteria should be limited to a pathological diagnosis based on en bloc resected specimens. In addition, full-thickness resection using transanal endoscopic surgery may be applied for cT1 rectal cancers in the case of technical difficulty in ER.

Being a retrospective study and having a small sample size are limitations of this study. Judgement of additional surgery for high-risk pT1 CRC was dependent on the patients’ or physicians’ choice, which would result in some confounding factors. Therefore, the present study focused on the high-risk pT1 CRC with surgery group to eliminate the bias. As the results, our data showed that the omission of completion surgery in all patients with pT1 CRC who have a risk factor of only SM invasion may present a significant risk. As we proposed in our novel criteria, application to more limited populations may be appropriate as the first step.

## 5. Conclusions

In conclusion, the present study shows that lymphatic and vascular invasions were potential independent risk factors for LNM, and rectal cancer and undifferentiated histology were independent risk factors for poor RFS in patients with high-risk pT1 CRC. Surgical treatment with lymph node dissection may be skipped for patients with high-risk pT1 CRC who meet our proposed criteria.

## Figures and Tables

**Table 1:** Patients’ characteristics.

		Low-Risk	High-Risk with ER Alone	High-Risk with Surgery	
		(*n* = 79)	(*n* = 40)	(*n* = 285)	p *
Age (years)	Median (range)	70 (37–86)	73 (40–87)	69 (34–90)	0.313
Gender	Male Female	45 34	21 19	154 131	0.855
Location	Colon Rectum	74 5	32 8	232 53	0.831
Tumor size (mm)	Median (range)	20 (4–53)	15 (6–58)	20 (5–130)	0.039
Morphology	Protruded type	43	28	131	0.002
	Flat or flat elevated type	14	9	53	
	Depressed type	22	3	101	
Pathological risk factors	Undifferentiated histology	0	0 (0%)	3 (1.1%)	>0.999
	SM invasion depth ≥1000 µm	0	37 (92.5%)	270 (94.7%)	0.473
	Vascular invasion	0	1 (2.5%)	59 (20.7%)	0.005
	Lymphatic invasion	0	5 (12.5%)	121 (42.5%)	<0.001
	Budding	0	2 (5.0%)	72 (25.3%)	0.004
Resection	ER alone	55	40	0	<0.001
	ER followed by curative surgery	7	0	102	
	Curative surgery	17	0	183	
Horizontal margin Vertical margin	HM0/HM1/HMX VM0/VM1/VMX	62/0/0 62/0/0	35/2/3 38/1/1	76/10/16 71/21/10	0.182 0.003
Number of examined lymph nodes Median (range)		4 (0–33)	−	9 (0–49)	−
Lymph node metastasis		1/24 (1.3%)	−	32/285 (11.2%)	−
Recurrence	Local intraluminal	0/79 (0%)	1/40 (0.3%)	0/285 (0%)	>0.999
	Local or distant metastasis	0/79 (0%)	0/40 (0%)	5/285 (1.8%)	>0.999

Low-risk, lesions without any pathological risk factors; High-risk, lesions with any pathological risk factors; SM invasion depth, submucosal invasion depth; ER, endoscopic resection. * High-risk with ER alone vs. high-risk with surgery.

**Table 2:** Risk factors for lymph node metastasis in the high-risk group.

		Lymph Node Metastasis		*p*	OR [95% CI]	*p*
		LNM (−) (*n* = 253)	LNM (+) (*n* = 32)			
Age	≤70 years	132 (86.3%)	21 (13.7%)	0.151		
	>70 years	121 (91.7%)	11 (8.3%)			
Gender	Male	135 (87.7%)	19 (12.3%)	0.520		
	Female	118 (90.1%)	13 (9.9%)			
Location	Colon	207 (89.2%)	25 (10.8%)	0.613		
	Rectum	46 (86.8%)	7 (13.2%)			
Tumor size	≤20 mm	168 (88.4%)	22 (11.6%)	0.791		
	>20 mm	85 (89.5%)	10 (10.5%)			
Morphology	Protruded	117 (89.3%)	14 (10.7%)	0.221		
	Flat/Flat elevated	50 (94.3%)	3 (5.7%)			
	Depressed	86 (85.1%)	15 (14.9%)			
Histology	Differentiated	252 (89.4%)	30 (10.6%)	0.034	1	0.071
	Undifferentiated	1 (33.3%)	2 (66.7%)		1.17 (0.81–166.7)	
SM invasion depth	≤2000 µm	73 (89.0%)	9 (11.0%)	>0.999		
	>2000 µm	180 (88.7%)	23 (11.3%)			
Vascular invasion	(−)	208 (92.0%)	18 (8.0%)	0.001	1	0.037
	(+)	45 (76.3%)	14 (23.7%)		2.42 (1.06–5.56)	
Lymphatic invasion	(−)	154 (93.9%)	10 (6.1%)	0.001	1	0.024
	(+)	99 (81.8%)	22 (18.2%)		2.64 (1.14–6.09)	
Budding	(−)	193 (90.6%)	20 (9.4%)	0.091		
	(+)	60 (83.3%)	12 (16.7%)			
Horizontal margin *	HM0	68 (89.5%)	8 (10.5%)	0.110		
	HM1/X	26 (100%)	0 (0%)			
Vertical margin *	VM0	67 (94.4%)	4 (5.6%)	0.241		
	HM1/X	27 (87.1%)	4 (12.9%)			
Number of examined LN	<12	170 (90.4%)	18 (9.6%)	0.218		
	≥12	83 (85.6%)	14 (14.4%)			

LNM, lymph node metastasis; SM invasion depth, submucosal invasion depth; OR, odds ratio; 95% CI, 95% confidential interval; HM, horizontal margin; VM, vertical margin. * Total number that assessed vertical and horizontal margins consisted of 104 patients with endoscopic resection before surgery.

**Table 3:** According to the stratification of risk factors, incidence of lymph node metastasis in the high-risk group.

* Positive or Unclear Vertical Margin	Total (*n* = 285)			
(1) SM Invasion Depth ≥1000 µm				
(2) Lymphovascular Invasion				
(3) Budding		Lymph Node Metastasis		Probability of LNM
(4) Undifferentiated Histology	*n*	LNM (−)	LNM (+)	
(1)	102	97	5	4.9%
(1) *	18	16	2	11.1%
(1) + (2)	82	70	12	14.6%
(1) + (3)	24	23	1	4.2%
(1) + (2) + (3)	41	32	9	21.4%
(1) + (2) + (3) + (4)	2	0	2	100%
				
(1) + (4)	0	−	−	−
(1) + (2) + (4)	0	−	−	−
(1) + (3) + (4)	1	1	0	0%
(2)	9	8	1	11.1%
(2) *	2	2	0	0%
(3)	2	2	0	0%
(4)	0	−	−	−
(2) + (3)	2	2	0	0%
(2) + (4)	0	−	−	−
(3) + (4)	0	−	−	−
(2) + (3) + (4)	0	−	−	−

LNM, Lymph node metastasis; SM invasion depth, submucosal invasion depth. Cases combining risk factors include cases with positive vertical margin.

**Table 4:** Risk factors for relapse-free survival in the high-risk group with surgical resection.

		Univariate Analysis		Multivariate Analysis	
		5-Year RFS	*p*	HR (95% CI)	*p*
Age	≤70 years	92.4%	0.536		
	>70 years	94.0%			
Gender	Male	91.6%	0.061		
	Female	94.8%			
Location	Colon	95.1%	0.032	1	0.049
	Rectum	85.5%		2.91 (1.01–8.40)	
Tumor size	≤20 mm	95.4%	0.402		
	>20 mm	88.5%			
Morphology	Protruded	97.7%	0.041		
	Flat/Flat elevated	97.2%			
	Depressed	87.1%			
Histology	Differentiated	93.6%	0.005	1	0.035
	Undifferentiated	50.0%		9.43 (1.17–7.69)	
SM invasion depth	≤2000 µm	95.5%	0.317		
	>2000 µm	92.4%			
Vascular invasion	(−)	94.0%	0.134		
	(+)	90.0%			
Lymphatic invasion	(−)	96.5%	0.257		
	(+)	89.2%			
Budding	(−)	94.3%	0.425		
	(+)	90.3%			
Horizontal margin *	HM0	95.0%	0.956		
	HM1/X	100%			
Vertical margin *	VM0	97.9%	0.267		
	HM1/X	95.0%			
Number of examined LN	<12	92.1%	0.771		
	≥12	96.1%			
Lymph node metastasis	(−)	93.8%	0.680		
	(+)	87.7%			

SM invasion depth, submucosal invasion depth; HR, hazard ratio; 95% CI, 95% confidential interval; HM, horizontal margin; VM, vertical margin; LN, lymph node. * Total number that assessed vertical and horizontal margins consisted of 104 patients with endoscopic resection before surgery.

**Table 5:** Summary of recurrent cases.

Age, Gender	Location	Size (mm)	Morphology	Histology	SM Invasion Depth (µm)	V	Ly	BD	Treatment	LNM/Dissected LN (*n*)	Months to Recurrence	Recurrent Site	Treatment	Current Situation
60, Male	Rectum (Rb)	30	IIa + IIc	Moderate	2000	−	+	+	Surgical resection	1/17	47 m	Local LN	Surgery	Chemotherapy for distant metastasis
55, Male	Sigmoid colon	12	IIa + IIc	Moderate	2600	−	−	−	Surgical resection	0/10	18 m	Lung	Chemotherapy	Cancer death
66, Male	Rectum (Ra)	25	IIa + IIc	Well	6000	−	−	+	Surgical resection	0/10	56 m	Liver	Surgery	Chemotherapy for distant metastasis
61, Male	Rectum (Rb)	35	IIc + IIa	Poorly	6000	+	+	+	Surgical resection	4/9	30 m	Lung	Surgery	No recurrence
64, Female	Rectum (Rb)	20	IIa + IIc	Moderate	6000	+	+	+	Surgical resection	0/1	26 m	Lung	Surgery	No recurrence

SM invasion depth, submucosal invasion depth; V, vascular invasion; Ly, lymphatic invasion; BD, budding; LNM/Dissected LN (*n*), the number of lymph node metastasis/the number of dissected lymph node. −, negative; +, positive.

**Table 6:** New proposal that can skip completion surgery for the high-risk pT1 CRC.

	High-Risk CRC with Surgery (*n* = 285)	High-Risk CRC with and without Surgery (*n* = 325)
Fulfill with All Below Criteria	Lymph Node Metastasis	Recurrence
Differentiated adenocarcinoma	0/29	0/39
	0%	0%
V (−) and Ly (−)		
Colon cancer		
SM invasion depth ≤2000 µm		
Negative vertical margin		
**Validation Cohort**		
	**High-Risk CRC** **with Surgery** **(*n* = 52)**	**High-Risk CRC** **with and without Surgery** **(*n* = 64)**
**Fulfill with All Below Criteria**	**Lymph Node Metastasis**	**Recurrence**
Differentiated adenocarcinoma	0/8 0%	0/8 0%
V (−) and Ly (−)		
Colon cancer		
SM invasion depth ≤2000 µm		
Negative vertical margin		
**Total**	**0/37**	**0/47**
	**0%**	**0%**

SM invasion depth, submucosal invasion depth; V, vascular invasion; Ly, lymphatic invasion.

## Data Availability

The data presented in this study are available on request from the corresponding author (T.S.).

## Supplementary Materials

The following supporting information can be downloaded at: https://www.mdpi.com/article/10.3390/cancers14030822/s1, Figure S1: Overall survival between endoscopic resection alone and surgical resection, Figure S2: Relapse-free survival in the high-risk CRC with surgical resection, Figure S3: Overall survival in the high-risk CRC with surgical resection, Figure S4: Decision tree to predict lymph node metastasis and recurrence, Figure S5: Decision tree to predict lymph node metastasis and recurrence in the validation cohort, Table S1: Lymph node metastasis stratified according to submucosal invasion depth in the high-risk pT1 CRC, Table S2: Risk factors for overall survival in the high-risk group with surgical resection. Table S3: Validation cohort of the high-risk pT1 CRC.

## References

[1] Fitzmaurice C., Abate D., Abbasi N., Abbastabar H., Abd-Allah F., Abdel-Rahman O., Abdelalim A., Abdoli A., Abdollahpour I., Global Burden of Disease Cancer, Collaboration (2019). Global, Regional, and National Cancer Incidence, Mortality, Years of Life Lost, Years Lived With Disability, and Disability-Adjusted Life-Years for 29 Cancer Groups, 1990 to 2017: A Systematic Analysis for the Global Burden of Disease Study. JAMA Oncol..

[2] Hashiguchi Y., Muro K., Saito Y., Ito Y., Ajioka Y., Hamaguchi T., Hasegawa K., Hotta K., Ishida H., Ishiguro M. (2020). Japanese Society for Cancer of the Colon and Rectum (JSCCR) guidelines 2019 for the treatment of colorectal cancer. Int. J. Clin. Oncol..

[3] Watanabe T., Itabashi M., Shimada Y., Tanaka S., Ito Y., Ajioka Y., Hamaguchi T., Hyodo I., Igarashi M., Ishida H. (2012). Japanese Society for Cancer of the Colon and Rectum (JSCCR) guidelines 2010 for the treatment of colorectal cancer. Int. J. Clin. Oncol..

[4] Ueno H., Mochizuki H., Hashiguchi Y., Shimazaki H., Aida S., Hase K., Matsukuma S., Kanai T., Kurihara H., Ozawa K. (2004). Risk factors for an adverse outcome in early invasive colorectal carcinoma. Gastroenterology.

[5] Yoda Y., Ikematsu H., Matsuda T., Yamaguchi Y., Hotta K., Kobayashi N., Fujii T., Oono Y., Sakamoto T., Nakajima T. (2013). A large-scale multicenter study of long-term outcomes after endoscopic resection for submucosal invasive colorectal cancer. Endoscopy.

[6] Kitajima K., Fujimori T., Fujii S., Takeda J., Ohkura Y., Kawamata H., Kumamoto T., Ishiguro S., Kato Y., Shimoda T. (2004). Correlations between lymph node metastasis and depth of submucosal invasion in submucosal invasive colorectal carcinoma: A Japanese collaborative study. J. Gastroenterol..

[7] Nakadoi K., Tanaka S., Kanao H., Terasaki M., Takata S., Oka S., Yoshida S., Arihiro K., Chayama K. (2012). Management of T1 colorectal carcinoma with special reference to criteria for curative endoscopic resection. J. Gastroenterol. Hepatol..

[8] Yoshii S., Nojima M., Nosho K., Omori S., Kusumi T., Okuda H., Tsukagoshi H., Fujita M., Yamamoto H., Hosokawa M. (2014). Factors Associated with Risk for Colorectal Cancer Recurrence After Endoscopic Resection of T1 Tumors. Clin. Gastroenterol. Hepatol..

[9] Participants in the Paris Workshop (2003). The Paris endoscopic classification of superficial neoplastic lesions: Esophagus, stomach and colon. Gastrointest. Endosc..

[10] Shimura T., Sasaki M., Kataoka H., Tanida S., Oshima T., Ogasawara N., Wada T., Kubota E., Yamada T., Mori Y. (2007). Advantages of endoscopic submucosal dissection over conventional endoscopic mucosal resection. J. Gastroenterol. Hepatol..

[11] Japanese Society for Cancer of the C, Rectum (2019). Japanese Classification of Colorectal, Appendiceal, and Anal Carcinoma: The 3d English Edition [Secondary Publication]. J. Anus. Rectum. Colon..

[12] Okuda Y., Shimura T., Kato H., Yamada T., Hirata Y., Natsume M., Iwasaki H., Yamaguchi R., Sakamoto E., Takahashi S. (2020). Pathological impact of transanal colorectal tube for obstructive colorectal cancer. Surg. Endosc..

[13] Brierley J., Gospodarowicz M., Wittekind C. (2017). TNM Classification of Malignant Tumours.

[14] Yasue C., Chino A., Takamatsu M., Namikawa K., Ide D., Saito S., Igarashi M., Fujisaki J. (2019). Pathological risk factors and predictive endoscopic factors for lymph node metastasis of T1 colorectal cancer: A single-center study of 846 lesions. J. Gastroenterol..

[15] Vermeer N.C.A., Backes Y., Snijders H.S., Bastiaannet E., Liefers G.J., Moons L.M.G., Van De Velde C.J.H., Peeters K.C.M.J., the Dutch T1 Colorectal Cancer Working Group (2019). National cohort study on postoperative risks after surgery for submucosal invasive colorectal cancer. BJS Open.

[16] Kudo S.E., Ichimasa K., Villard B., Mori Y., Misawa M., Saito S., Hotta K., Saito Y., Matsuda T., Yamada K. (2021). Artificial Intelligence System to Determine Risk of T1 Colorectal Cancer Metastasis to Lymph Node. Gastroenterology.

[17] Yamashita K., Oka S., Tanaka S., Nagata S., Hiraga Y., Kuwai T., Furudoi A., Tamura T., Kunihiro M., Okanobu H. (2019). Preceding endoscopic submucosal dissection for T1 colorectal carcinoma does not affect the prognosis of patients who underwent additional surgery: A large multicenter propensity score-matched analysis. J. Gastroenterol..

[18] Ikematsu H., Yoda Y., Matsuda T., Yamaguchi Y., Hotta K., Kobayashi N., Fujii T., Oono Y., Sakamoto T., Nakajima T. (2013). Long-term Outcomes After Resection for Submucosal Invasive Colorectal Cancers. Gastroenterology.

[19] Kessels K., Backes Y., Elias S.G., Blink A.V.D., Offerhaus G.J.A., van Bergeijk J.D., Groen J.N., Seerden T.C., Schwartz M.P., Cappel W.H.D.V.T.N. (2019). Pedunculated Morphology of T1 Colorectal Tumors Associates with Reduced Risk of Adverse Outcome. Clin. Gastroenterol. Hepatol..

[20] Oka S., Tanaka S., Kanao H., Ishikawa H., Watanabe T., Igarashi M., Saito Y., Ikematsu H., Kobayashi K., Inoue Y. (2011). Mid-term prognosis after endoscopic resection for submucosal colorectal carcinoma: Summary of a multicenter questionnaire survey conducted by the colorectal endoscopic resection standardization implementation working group in Japanese Society for Cancer of the Colon and Rectum. Dig. Endosc..

[21] Backes Y., Elias S.G., Bhoelan B.S., Groen J.N., Van Bergeijk J., Seerden T.C.J., Pullens H.J.M., Spanier B.W.M., Geesing J.M.J., Kessels K. (2017). The prognostic value of lymph node yield in the earliest stage of colorectal cancer: A multicenter cohort study. BMC Med..

[22] Haasnoot K.J.C., Backes Y., Moons L.M.G., Kranenburg O., Trinh A., Vermeulen L., Noë M., Tuynman J.B., van Lent A.U.G., van Ginneken R. (2020). Associations of non-pedunculated T1 colorectal adenocarcinoma outcome with consensus molecular subtypes, immunoscore, and microsatellite status: A multicenter case-cohort study. Mod. Pathol..

[23] Naxerova K., Reiter J.G., Brachtel E., Lennerz J.K., van de Wetering M., Rowan A., Cai T., Clevers H., Swanton C., Nowak M.A. (2017). Origins of lymphatic and distant metastases in human colorectal cancer. Science.

[24] Schmoll H.J., Van Cutsem E., Stein A., Valentini V., Glimelius B., Haustermans K., Nordlinger B., van de Velde C.J., Balmaña J., Regula J. (2012). ESMO Consensus Guidelines for management of patients with colon and rectal cancer. A personalized approach to clinical decision making. Ann. Oncol..

[25] Benson A.B., Schrag D., Somerfield M.R., Cohen A.M., Figueredo A.T., Flynn P.J., Krzyzanowska M.K., Maroun J., McAllister P., Van Cutsem E. (2004). American Society of Clinical Oncology recommendations on adjuvant chemotherapy for stage II colon cancer. J. Clin. Oncol..

