# Supplementary material for: The Risk Analyses of Lymph Node Metastasis and Recurrence for Submucosal Invasive Colorectal Cancer: Novel Criteria to Skip Completion Surgery
Source: Cancers (Basel). 2022 Feb 6;14(3):822. doi: 10.3390/cancers14030822 (PMC8834028; doi:10.3390/cancers14030822)
Supplement: Supplementary file 1 [file cancers-14-00822-s001.zip › cancers-1554325-supplementary.pdf]

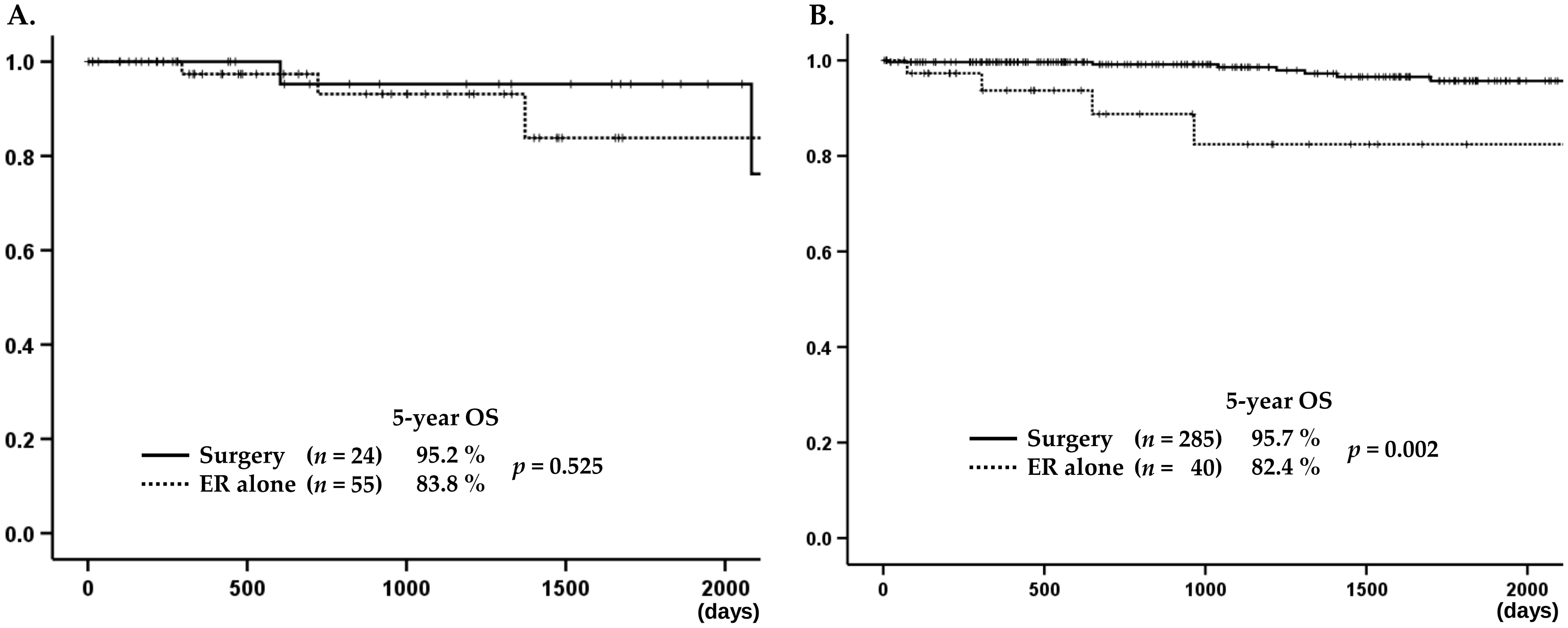

Supplementary Figure S1. Overall survival between endoscopic resection alone and surgical resection
A. Low-risk pT1, B. High-risk pT1
ER, endoscopic resection; 5-year OS, 5-year overall survival rate

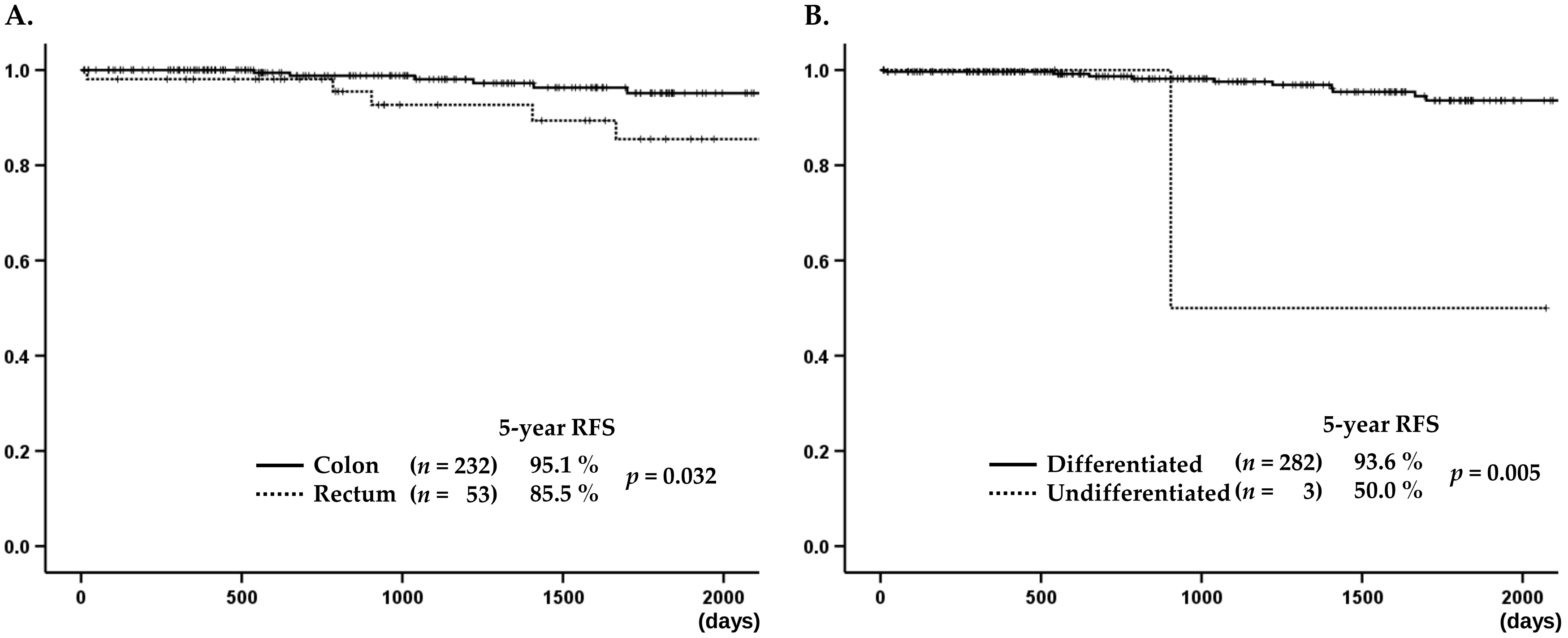

Supplementary Figure S2. Relapse-free survival in the high-risk CRC with surgical resection
A. Tumor location, B. Histology
5-year RFS, 5-year relapse-free survival rate

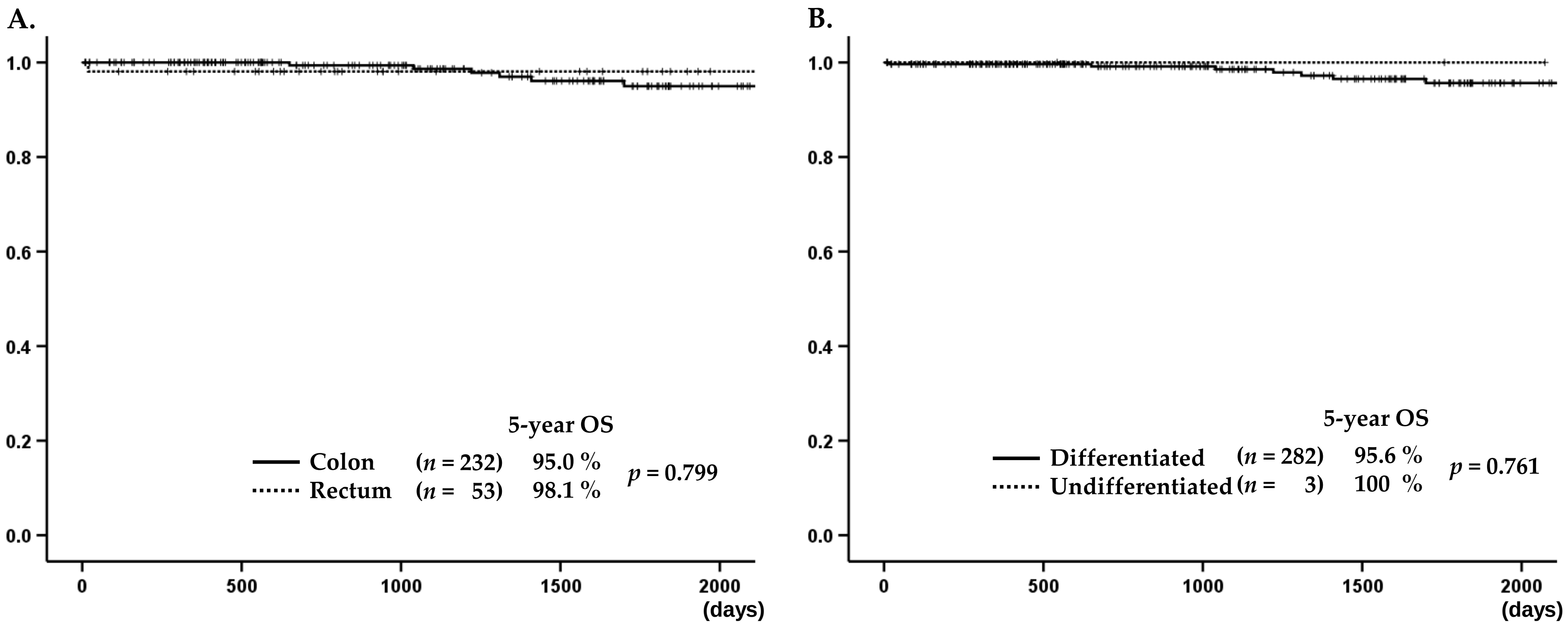

Supplementary Figure S3. Overall survival in the high-risk CRC with surgical resection
A. Tumor location, B. Histology
5-year OS, 5-year overall survival rate

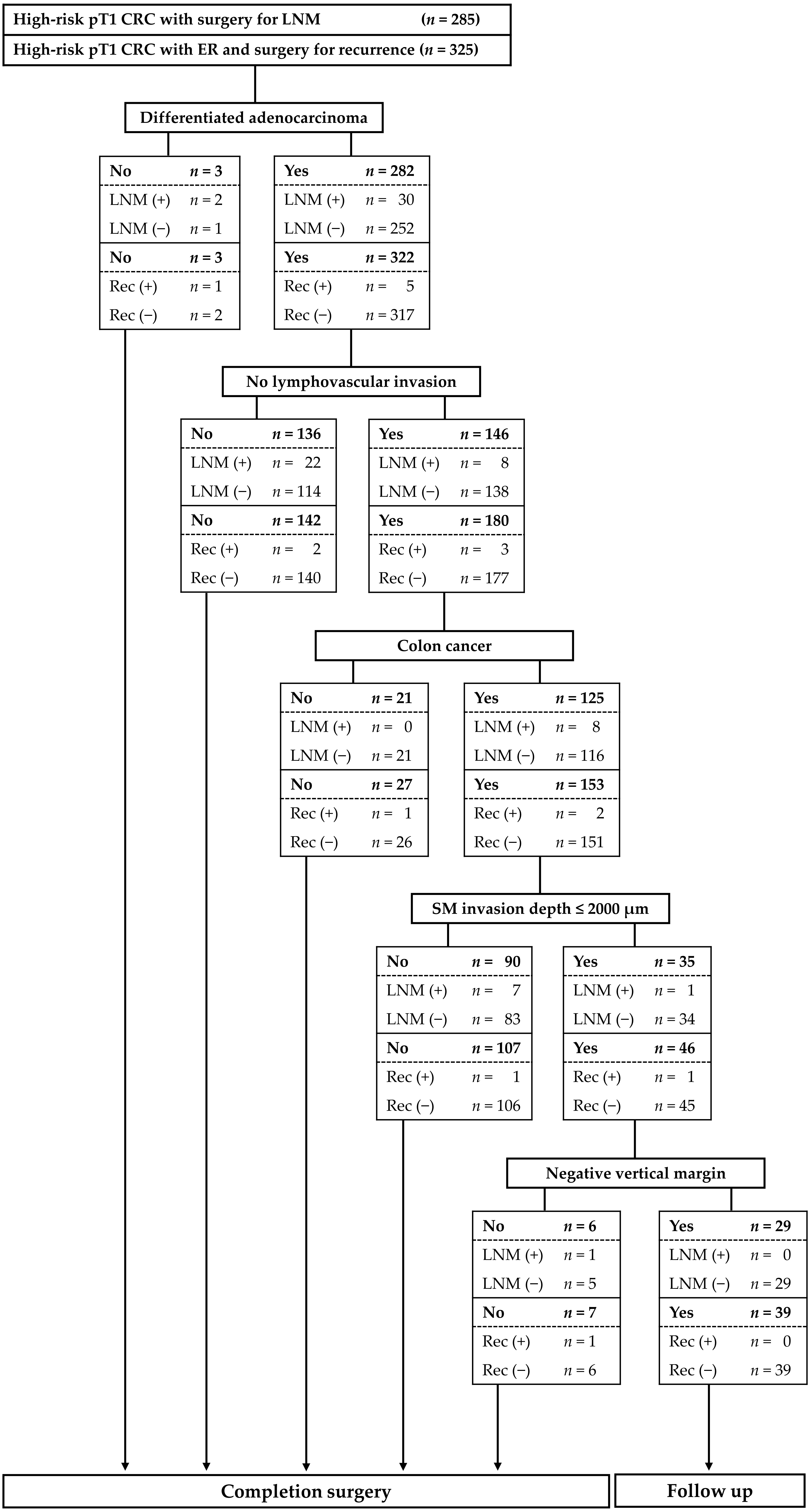

Supplementary Figure S4. Decision tree to predict lymph node metastasis and recurrence
The incidence of LNM was analyzed using the high-risk pT1 CRC with surgery group **(*n* = 285)**.
The incidence of recurrence was analyzed using all high-risk pT1 CRCs with ER and surgery **(*n* = 325)**.
ER, endoscopic resection; LNM, lymph node metastasis; Rec, recurrence including metastatic and local intraluminal recurrences

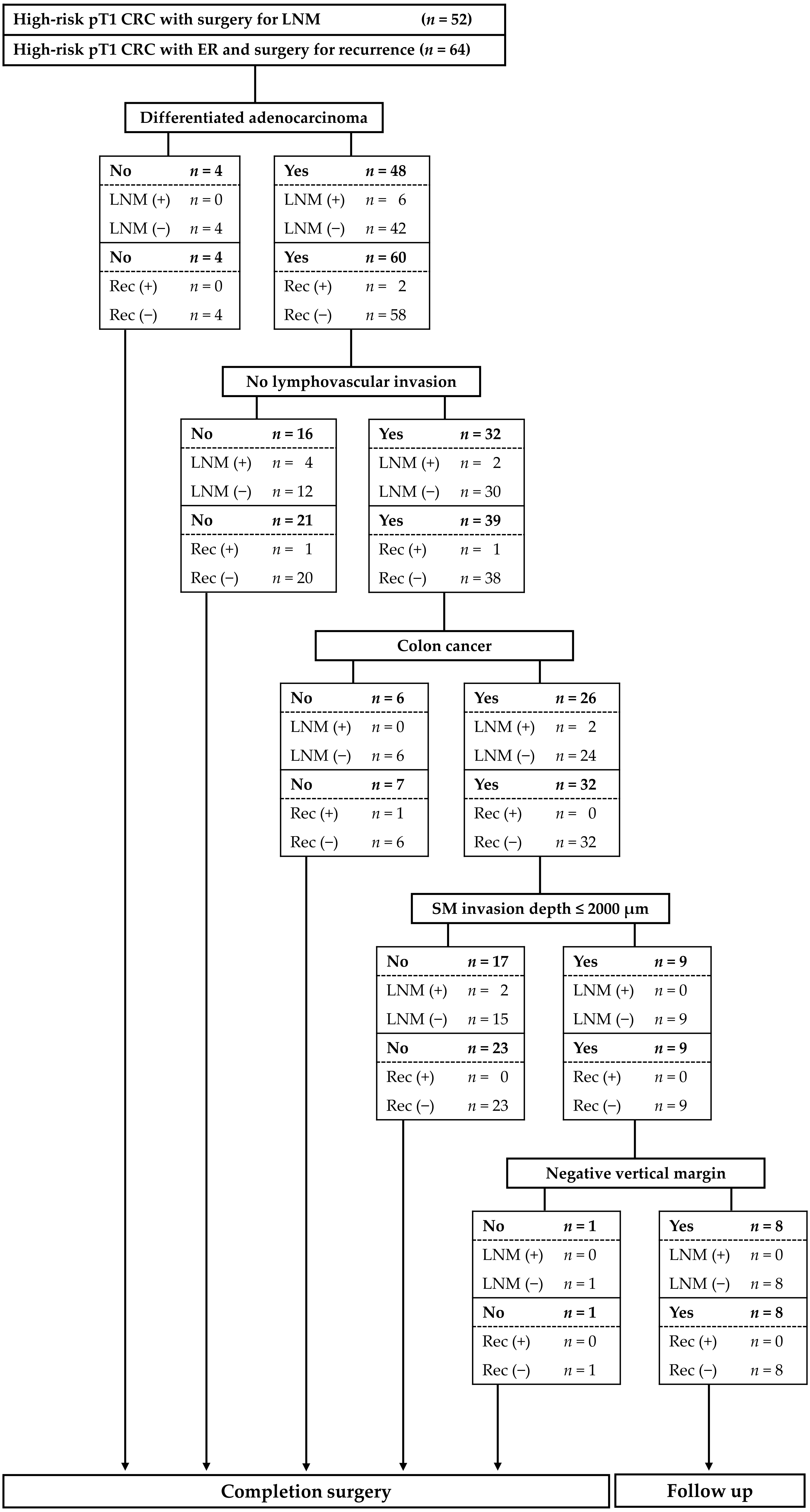

Supplementary Figure S5. Decision tree to predict lymph node metastasis and recurrence in the validation cohort
The incidence of LNM was analyzed using the high-risk pT1 CRC with surgery group **(*n* = 52)**.
The incidence of recurrence was analyzed using all high-risk pT1 CRCs with ER and surgery **(*n* = 64)**.
ER, endoscopic resection; LNM, lymph node metastasis; Rec, recurrence including metastatic and local intraluminal recurrences

Supplementary Table S1. Lymph node metastasis stratified according to submucosal invasion depth in the high-risk pT1 CRC

Risk factor: SM invasion depth alone	*n*	Lymph node metastasis		Incidence of LNM
		LNM (−)	LNM (+)	
1000 μm ≤ SM ≤ 2000 μm	23	23	0	0 %
2000 μm < SM ≤ 3000 μm	26	24	2	7.7 %
3000 μm < SM ≤ 4000 μm	24	22	2	8.3 %
4000 μm ≤ SM	29	28	1	3.4 %
Total	102	97	5	4.9 %

Lesions with negative vertical margin that had only SM invasion depth as a risk factor were analyzed.
LNM, Lymph node metastasis; SM, submucosal invasion depth

Risk factor: VM (+)	*n*	Lymph node metastasis		Incidence of LNM
		LNM (−)	LNM (+)	
SM ≤ 2000 μm	5	4	1	25 %
2000 μm < SM ≤ 3000 μm	8	7	1	12.5 %
3000 μm < SM ≤ 4000 μm	2	2	0	0 %
4000 μm ≤ SM	3	3	0	0 %
Total	18	16	2	11.1%

Lesions with positive vertical margin were analyzed.
LNM, Lymph node metastasis; SM, submucosal invasion depth; VM (+), positive vertical margin

Risk factor: V/Ly (+) +VM (−)	*n*	Lymph node metastasis		Incidence of LNM
		LNM (−)	LNM (+)	
SM ≤ 2000 μm	33	28	5	16.7 %
2000 μm < SM ≤ 3000 μm	20	17	3	15.0 %
3000 μm < SM ≤ 4000 μm	11	9	2	18.2 %
4000 μm ≤ SM	22	20	2	9.1 %
Total	86	74	12	14.0 %

Lesions with negative vertical margin that had lymphovascular invasion as a risk factor were analyzed.
LNM, Lymph node metastasis; SM, submucosal invasion depth; V/Ly (+), positive lymphovascular invasion; VM (−), negative vertical margin

Risk factor: BD (+) + VM (−)	*n*	Lymph node metastasis		Incidence of LNM
		LNM (−)	LNM (+)	
SM ≤ 2000 μm	8	8	0	0 %
2000 μm < SM ≤ 3000 μm	9	8	1	11.1 %
3000 μm < SM ≤ 4000 μm	3	3	0	0 %
4000 μm ≤ SM	5	5	0	0 %
Total	25	24	1	4.0 %

Lesions with negative vertical margin that had budding as a risk factor were analyzed.
LNM, Lymph node metastasis; SM, submucosal invasion depth; BD (+), positive budding; VM (−), negative vertical margin

Supplementary Table S2. Risk factors for overall survival in the high-risk group with surgical resection

		Univariate analysis	
		**5-year OS**	***p***
Age	≤70 years	96.8 %	0.768
	>70 years	94.1 %	
Gender	Male	95.0 %	0.072
	Female	96.5 %	
Location	colon	95.0 %	0.799
	rectum	98.1 %	
Tumor size	≤20 mm	95.9 %	0.698
	>20 mm	95.3 %	
Morphology	Protruded	96.1 %	0.600
	Flat/Flat elevated	97.2 %	
	Depressed	94.9 %	
Histology	differentiated	95.6 %	0.761
	undifferentiated	100 %	
SM invasion depth	≤2000 μm	98.4 %	0.211
	>2000 μm	95.48%	
Vascular invasion	(−)	95.8 %	0.470
	(+)	95.0 %	
Lymphatic invasion	(−)	97.7 %	0.757
	(+)	93.2 %	
Budding	(−)	94.1 %	0.193
	(+)	100 %	
Horizontal margin*	HM0	95.1 %	0.442
	HM1/X	93.8 %	
Vertical margin*	VM0	97.9 %	0.111
	HM1/X	90.0 %	
Number of dissected LN	<12	94.7 %	0.662
	≥12	99.0 %	
Lymph node metastasis	(−)	95.2 %	0.195
	(+)	100 %	

SM invasion depth, submucosal invasion depth

HM, Horizontal margin; VM, vertical margin; LN, lymph node

*, Total number that assessed vertical and horizontal margins was consisted of 104 patients with endoscopic resection before surgery.

Supplementary Table S3. Validation cohort of the high-risk pT1 CRC

		High-risk with ER alone (n = 12)	High-risk with surgery (n = 52)
Age (years)	Median (range)	76 (45-92)	65 (25-87)
Gender	Male	7	32
	Female	5	20
Location	Colon	10	41
	Rectum	2	11
Tumor size (mm)	Median (range)	11 (8-25)	18 (6-35)
Morphology	Protruded type	9	30
	Flat or Flat elevated type	0	0
	Depressed type	3	22
Pathological risk factors	Undifferentiated histology	0 (0 %)	4 (7.7 %)
	SM invasion depth ≥1000 μm	12 (100 %)	48 (92.3 %)
	Vascular invasion	1 (8.3 %)	9 (17.3 %)
	Lymphatic invasion	4 (33.3 %)	12 (23.1 %)
	Budding	1 (8.3 %)	11 (21.2 %)
Surgical resection	ER alone	12	0
	ER followed by curative surgery	0	25
	Curative surgery	0	27
Horizontal margin	HM0/HM1/HMX	12/0/0	24/0/1
Vertical margin	VM0/VM1/VMX	12/0/0	20/4/1
Number of examined lymph nodes	Median (range)	–	15 (2-47)
Lymph node metastasis		–	6/52 (11.5%)
Recurrence	local	0/12 (0 %)	0/52 (0 %)
	metastasis	0/12 (0 %)	2/52 (3.8 %)

High-risk, lesions with any of pathological risk factors; SM invasion depth, submucosal invasion depth; ER, endoscopic resection
